# Comparative Study of Smart Scope^®^ Visual Screening Test with Naked Eye Visual Screening and Pap Test

**DOI:** 10.31557/APJCP.2020.21.12.3509

**Published:** 2020-12

**Authors:** Veena Rahatgaonkar, Pooja Uchale, Gauri Oka

**Affiliations:** 1 *Department of Gynecology, Deenanath Mangeshkar Hospital and Research Centre, Pune, Maharashtra, India. *; 2 *Department of Research, Deenanath Mangeshkar Hospital and Research Centre, Pune, Maharashtra, India. *

**Keywords:** Cervical cancer, Pap test, VIA- VILI, Smart Scope® visual screening test, digital device

## Abstract

**Background:**

Cervical cancer is a major contributor to mortality and morbidity in women. Naked eye visual screening (NE test) and Pap test are commonly used for cervical cancer screening. Both tests have inherent limitations like low sensitivity (Pap test) and subjectivity in interpretation, lack of permanent record and overestimation (NE test). Here, Smart Scope^®^ visual screening test (SS test) was compared with NE and Pap tests. Smart Scope^®^ is a small, hand-held device that captures cervical images attached to a tablet to store data.

**Objective:**

To compare SS test with Pap and NE tests.

**Study Design:**

This prospective observational study was conducted at a tertiary care hospital in India, over 16 months. A total of 509 women in the age group of 25 to 65 years were included in the study as per the inclusion criteria. All the participants underwent Pap test, NE test and SS test. Screen positives on any one test were advised colposcopy and biopsy.

**Results:**

Out of 154 screen-positive women, 49 visited for follow-up colposcopy-guided biopsy. Nine incidental biopsies of screen-negative women were included in the data. Thus, statistical analysis was carried out based on 58 available histopathology results. Out of 58 biopsies, 8 were normal, 30 were benign lesions, 18 were precancerous and 2 were cancerous lesions. SS test was found to have a sensitivity and NPV of 100% each, PPV of 45.4% and a specificity of 36.8%. Sensitivity and specificity of NE test was 90% and 39.5% respectively, PPV was 43.9% and NPV was 88.2%. Pap smear had a sensitivity of 25% and specificity of 84.2%, PPV of 45.5% and NPV of 68.08%.

**Conclusion:**

SS test has great potential to be a primary screening test in low-resource settings due to its better sensitivity and NPV as compared to NE and Pap tests.

## Introduction

Cervical cancer is a major contributor to mortality and morbidity among women in low- and middle-income countries (Ferlay et al., 2019). Unlike other cancers, cervical cancer is preceded by a spectrum of cyto-morphological changes called cervical intraepithelial neoplasia (CIN) for many years before developing into a frank malignancy. If the disease is detected in this pre-invasive stage by screening, conservative treatment can be offered and progression to frank malignancy can be avoided (WHO, 2014). Well-organized and well-implemented cervical cytology programs in high-resource countries have reduced the mortality by 20-60% (Hakama et al., 1985; Antal et al., 1986; Stjernsward et al., 1987; Miller et al., 1990). However, it has been reported that the disease burden is still high and 85% of the global mortality is seen in low-resource settings (WHO, 2019). 

Visual inspection with acetic acid (VIA) and visual inspection with Lugol’s iodine (VILI) are recommended by WHO as screening methods for cervical cancer in resource-limited countries. In India, the guidelines for community-based cervical screening programs based on VIA were formulated in the year 2005 (Govt. of India and WHO, 2006).Sensitivity and specificity of VIA were reported to be in the range of 67% - 79% and 49%- 86% respectively and those for VILI were 77.8% -98%, and 73% - 91.3%, respectively (Sankaranarayanan et al., 2005). However, visual screening tests have limitations like extreme subjectivity in interpreting tests, lack of permanent record, low reproducibility, overestimation and overtreatment.

Considering the various requirements of a screening test in a low-resource setting, a digital cervical screening test, namely, Smart Scope^®^ visual screening test (SS test) was developed. This is a Smart Scope^®^ (SS) aided visual screening method for detection of various cervical lesions. In this study, we have compared the efficacy of the SS test with naked eye screening test (NE test) and cervical cytology test (Pap test) for detection of precancerous and cancerous cervical lesions. 

## Materials and Methods

This prospective observational study was conducted at a multispecialty tertiary care hospital in western India over a period of 16 months from June 2018 to September 2019. A total of 509 women in the age group of 25 to 65 years visiting the cancer prevention clinic for screening were included in the study. Women were screened as per our inclusion criteria and consecutive consenting women were enrolled. Pregnant women, women with frank cervical growths and those who had undergone any cervical procedure within the last 8 weeks were excluded from the study. Prior to enrolment, the procedure was explained to the participant in detail.

*The Smart Scope* ^®^ *Device*

**Figure 1:**
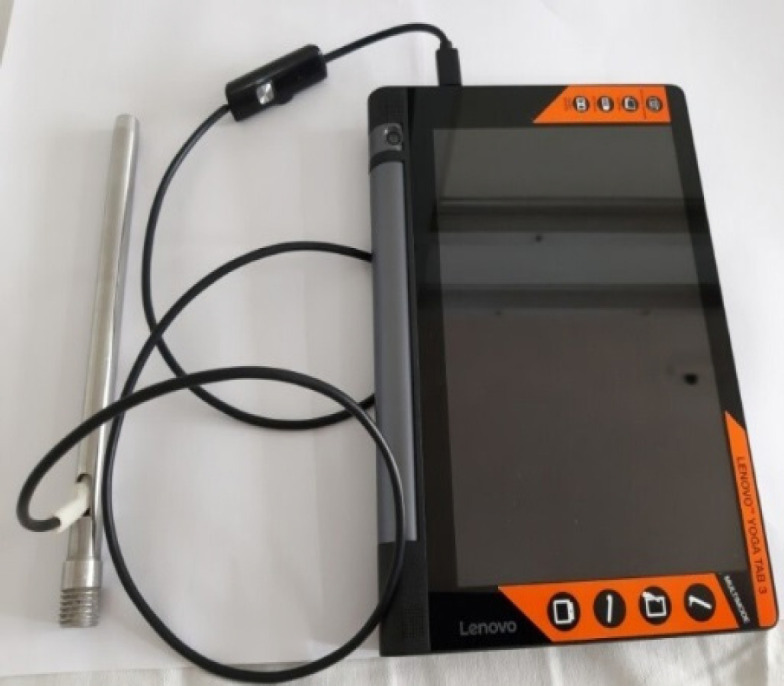
Smart Scope^®^ Device Attached to a Portable Computer

Smart Scope^®^ (model CX1.0, Periwinkle Technologies Pvt. Ltd., Pune, India) is a portable, 18 cm long stainless steel device with a 12 mm diameter (Figure 1). The vertical arm and optical end of the device is covered with a disposable sheath. The optical end is fitted with light sources and a camera having a focal length of 4 cm. It offers 5 x magnification. The device is attached to a portable computer in which Net4Medix^®^ software is installed for storage of images and relevant data. 

*Methodology*

The study was carried out in an out-patient setting where typically, patients from urban and rural areas, as well as, from different socio-economic strata come for regular check-up. Relevant clinical and obstetric history of participants was taken. Screening was carried out by a gynecologist. All the participants underwent Pap test (Liquid Based Cytology), NE test and SS test. Pap test (Papanicolaou and Traut, 1941) and NE test (Sankaranarayanan and Wealey, 2003) were performed as per standard protocols. 

**Figure 2:**
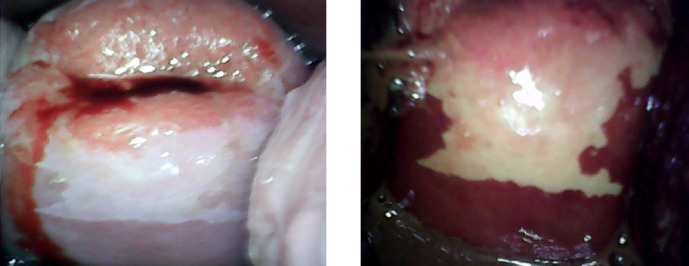
Smart Scope^®^ Screening Test: Positive

**Figure 3:**
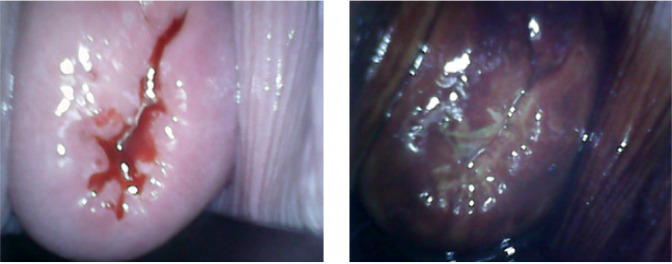
Smart Scope® Screening Test: Negative

**Figure 4:**
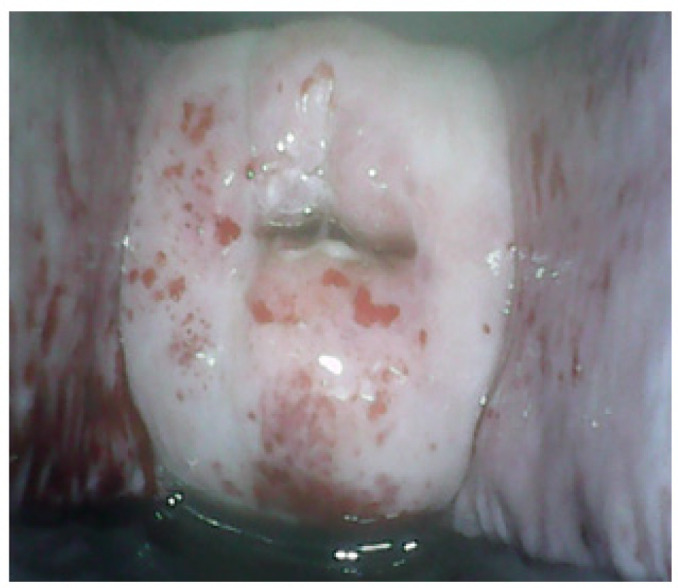
Smart Scope^®^ Screening Test: Unsatisfactory

The cervix was cleaned with normal saline. The Pap smear was taken. Women identified with atypical squamous cells of undetermined significance (ASCUS), atypical squamous cells, cannot exclude HSIL (ASC-H), low-grade intraepithelial lesion (LSIL), high-grade intraepithelial lesion (HSIL) and cancerous (Ca) on Pap test were considered as positive. One minute after application of 5% acetic acid to cervix, NE test findings were noted. Smart Scope^®^ was inserted in the vagina and the cervical image was taken. Lugol’s iodine was applied to cervix and NE test and SS test findings were recorded. A well-demarcated aceto-white area (AWA) or iodine-negative area abutting the squamo-columnar junction was considered as VIA or VILI positive respectively (Figure 2). Absence of definite AWA or iodine-negative area was considered as negative result (Figure 3). If the squamo-columnar junction (SCJ) was not seen or the cervical view was obscured by inflammation or bleeding, it was categorized as an unsatisfactory result (Figure 4).Women screened positive on any of the above tests, were advised a colposcopy and a colposcopy-guided biopsy, if required. Histopathology results were considered as gold standard. Nine participants were found which were negative on all the three tests, but had undergone hysterectomy for benign indications. Cervical histopathology reports of these women were available and were included in the study as they provided incidental biopsy data of negative results of the three screening tests.

*Data analysis*

Statistical analysis was carried out with the help of SPSS (version 23) for Windows package (SPSS Science, Chicago, IL, USA). Sensitivity, specificity, negative predictive value (NPV) and positive predictive value (PPV) for Pap test, NE test and SS test were calculated. Chi-square test was used for examining the association between the histopathology and the three screening tests. A p value of less than 0.05 was considered as significant.

*Ethical considerations*

Approval (IHR_2018_APR_VR_265) of Institutional Ethics Committee (DCGI Reg. No. ECR/15/Inst/Maha/2013/RR-19) was obtained prior to initiation of the study. 

## Results

Out of 509 participants, maximum women were in the age group of 31 to 40 years (36%). The mean age of participants was 38.9± 9.9years. As seen in Table 1, Pap test could detect 1 HSIL and 8 LSIL. Maximum number of women, (300/509; 58.9%), were reported to have normal cytology followed by 169 (33.2%) with inflammatory smears. On NE test, 141 were screen-positive (27.7%), and 367 were screen-negative (72.1%). SS test identified pre-cancerous lesions in 94 (18.5%) and cancerous lesions in 2 (0.4%) women. 

**Figure 5:**
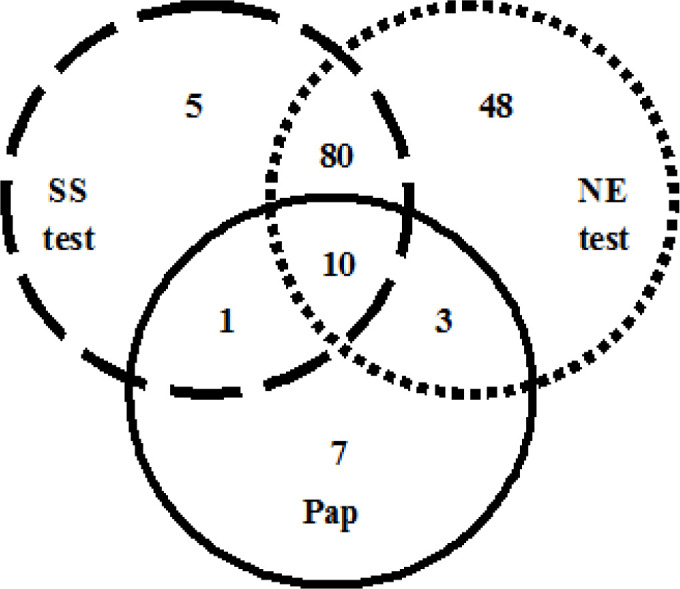
Distribution of 154 Screen-Positive Women in Three Screening Tests. SS test, Smart Scope visual screening test; NE test, Naked eye visual screening test; Pap, Pap smear test

One hundred and fifty-four women were found positive on at least one of the three screening tests (Fig 5). They were advised colposcopy and biopsy. Out of these, only 49 women came back for the biopsy. The rest were lost to follow-up. Biopsy reports of 9 women who had tested negative on screening by all the three tests were incidentally available as they had undergone hysterectomies for various indications. These reports were added to our data of 49 women. Thus, data of 58 women is presented. Out of these, 18 women were diagnosed with precancerous lesions and 2 with cancer on histopathology. 

On comparing 11 precancerous Pap results with the corresponding histopathology outcomes, it was seen that 2 out of 4 LSIL on Pap were CIN I (Table 2). One HSIL was micro-invasive malignancy. Out of 5 ASCUS on Pap, 1 was CIN I and 1 was CIN II. In all, 10 CIN I, 2 CIN II, 1 CIN III and 1 cancer case were missed on Pap smear. 1 CIN I on histology was reported as unsatisfactory on Pap smear.The sensitivity, specificity, PPV and NPV of Pap test were 25%, 84.2%, 45.5% and 68.08% respectively.

Out of 41 biopsy results available for positive NE test results, 16 were confirmed precancerous and 2 as cancerous cases (Table 2) indicating 90% sensitivity. However, NE test missed 2 precancerous cases. The specificity, PPV and NPV of NE test was 39.5%, 43.9% and 88.2%respectively (p=0.02).

Out of 44 biopsies available for positive SS test (Table 2), 18 were precancerous and 2 were cancerous lesions. Out of 18 precancerous lesions detected by SS test, 14 were diagnosed as CIN I, 3 as CIN II and 1 as CIN III on histopathology. There was a significant association between the results obtained by SS test and histopathology (p=0.002).The sensitivity and NPV of SS test were 100% each, specificity 36.8% and PPV was 45.4%. 

**Table 1:** Distribution of Results of Pap Test, NE Test and SS Test (N=509)

Screening Test	Result		Total (%)
Pap Test	Normal		300 (58.94)
	Benign	Atrophic	3 (0.59)
		Inflammation	169 (33.2)
	Pre-cancerous	ASCUS	11 (2.16)
		ASC-H	1 (0.2)
		LSIL	8 (1.57)
		HSIL	1 (0.2)
	Unsatisfactory		16 (3.14)
NE test	Negative		367 (72.1)
	Positive		141 (27.7)
	Unsatisfactory		1 (0.2)
SS test	Negative		406 (79.76)
	Positive	Pre-Ca	94 (18.47)
		Ca	2 (0.39)
	Unsatisfactory		7 (1.38)

ASCUS, Acronym for Atypical Squamous Cells of Undetermined Significance; ASC-H, Atypical squamous cells cannot exclude HSIL; CA, cancerous; HSIL, high-grade intraepithelial lesion; LSIL, low-grade intraepithelial lesion; NE test, Naked eye visual screening test; SStest, Smart Scope® visual screening test

**Table 2:** Distribution of Histopathological Outcome (n=58)

Screening Tests			Histopathology Result					
		n	Normal	Benign	CIN I	CIN II	CIN III	Ca
		58	8	30	14	3	1	2
Pap								
Normal		28	3	18	6	0	1	0
Benign		17	4	6	4	2	0	1
Pre-cancerous	ASCUS	5	0	3	1	1	0	0
	ASC-H	1	1	0	0	0	0	0
	LSIL	4	0	2	2	0	0	0
	HSIL	1	0	0	0	0	0	1
Unsatisfactory		2	0	1	1	0	0	0
NE test								
Negative		17	6	9	1	0	1	0
Positive		41	2	21	13	3	0	2
Unsatisfactory		0	0	0	0	0	0	0
SS test								
Negative		14	6	8	0	0	0	0
Positive	Pre-Ca	42	2	22	14	3	1	0
	Ca	2	0	0	0	0	0	2
Unsatisfactory		0	0	0	0	0	0	0

ASCUS, Acronym for Atypical Squamous Cells of Undetermined Significance; ASC-H, Atypical squamous cells cannot exclude HSIL; CA, cancerous;CIN, cervical intraepithelial neoplasia;HSIL, high-grade intraepithelial lesion; LSIL, low-grade intraepithelial lesion; NE test, Naked eye visual screening test; SStest, Smart Scope® visual screening test

## Discussion

Historically, several researchers have compared the performances of various screening tests throughout the world (Sankaranarayanan et al., 2005; Pimple et al., 2010; Karimi-Zarchi et al., 2013; Bhattacharyya et al., 2015; Davies et al., 2015; Fokom-Domgue et al., 2015; Pourasad-Shahrak et al., 2015; Qiao et al., 2015; Ami and Singh, 2016; Bobdey et al., 2016). Well-implemented screening programs using Pap smear test have found to reduce the mortality and incidence of cervical cancer in Western European countries and the USA (Stjernsward et al., 1987). On the other hand, implementation of such screening programs failed in resource-limited settings (Stjernsward et al., 1987). Also contributory were some inherent drawbacks of Pap screening such as high cost, limited availability of cytology laboratories and repeated visits required for report collection. Human papilloma virus (HPV) DNA test, though highly sensitive, is not recommended for women below 30 years of age (Chelmow, 2016). The high cost and need of specialized molecular laboratory set up are the main downsides for the successful implementation of HPV-based screening programs in resource-limited settings. 

The visual screening method is a low-cost approach for cervical cancer screening. Though it has demonstrated encouraging results on cervical cancer prevention as indicated by various studies including those conducted by WHO and International Agency for Research on Cancer (Sankaranarayanan et al., 2004; Sankaranarayanan et al., 2007; Sauvaget et al., 2011; Government of India, 2019), it has its own limitations such as subjectivity in interpretation of results, lack of standardization, requirement of frequent training of health care providers, overestimation and overtreatment, and low reproducibility due to lack of permanent record. Due to the subjective nature of interpretation of results, sensitivity and specificity of VIA for CIN II+ varies between 66.5–80.0% and 82.9–90.4%, respectively and those for VILI in the range of 81.5–94.7% and 81.7–90.0%, respectively (Qiao et al., 2015). Researchers have used a magnifying glass as a low-cost inspection tool in visual screening (VIAM) (Parashari et al., 2000; Singh et al., 2014). In VIAM the light source is kept outside the vagina, making it impossible for the maneuvering of vision to observe the suspected area closely. 

The results of the SS test for cervical cancer screening have not been published before. Smart Scope^®^ is a non-invasive, portable, device which is easy to operate. The tip of the device is kept 4cm away from the cervix, and is fitted with a light source, thereby allowing a close and magnified view of the cervix with better clarity and maneuverability. As the software Net4Medix^®^ of the device keeps a digital log of the images, it becomes useful at the time of follow up visits. Due to this advantage, images captured at a remote heath care center could be easily transferred to higher centers for expert analysis. The sensitivity of the SS test at CIN I + threshold is 100% with a NPV of 100%. 

Recently, Bedell et al., (2020) summarized various methods including new technologies for screening and treatment of cervical cancers and pre-cancers. Apart from Smart Scope^®^, currently, there is only one intra-vaginal device based on optical camera image processing technology, namely, Pocket colposcope (Lam et al., 2015; Lam et al., 2018). In 2018, Mueller et al., (2018) published a study where digital images of 129 “pre-identified abnormal cytology and/or HPV positive” women were considered for comparison with the performance of Pocket colposcope (sensitivity 71.2%, specificity 57.5%) with that of standard colposcope (sensitivity 79.8%, specificity 56.6%). 

TruScreen^TM^ is an intravaginal portable optoelectronic screening device. The efficacy of this device was studied in many countries including China, Poland, and Russia. It is shown to be better than Pap test and has 63 to 100% sensitivity at different CIN I+ thresholds (Sukhikh et al., 2009; Pruski et al., 2011; Özgü et al., 2015; Yang et al., 2018). The tip of the scanner containing biosensors touches the cervix during examination and hence is disposable, making it an expensive proposition. 

In 2012, Bae et al., (2012) assessed the utility of a 35mm camera to record cervical images. In this retrospective study, agreement rates between cervicography and pathology in CIN I, CIN II or III, and cervical cancer were 52.0%, 78.9%, and 90.2%, respectively. 

Enhanced Visual Assessment (EVA), a smartphone enabled colposcope developed by Mobile ODT, Israel, could accurately differentiate between CIN I and CIN II+ lesions in 30 high risk HPV positive Cambodian women (Thay et al., 2019). Another pilot trial using a smartphone for diagnosis of pre-cancer in 20 patients with abnormal cervical cytology could identify 85% pathologically confirmed CIN I+ cases (Tanaka et al., 2017). This study was undertaken to evaluate “diagnostic” capability of the device but the sample size in this study was too small to comment conclusively. Bhatt et al., (2018) tried to employ mobile technology coupled with a dedicated application for capturing images and tele-analysis in rural parts of India in 2018. Not many women responded to their initiative. Only 170 women opted for cervical screening and out of those, only 18 came for follow-up. In our study, the lost-to-followup rate among screen positives was high. In a country like India, it is a common scenario that the follow up rate of women is low due to various factors like fear of cancer, socio-economic constraints, and lack of family support. Our experience is that screen-negative women are less likely to return for an invasive procedure like biopsy. Therefore, we recommended biopsy only for the screen-positive women.

Gynocular^TM^, a portable colposcope, was evaluated for its efficacy as a triaging method for VIA/HPV positive women, in Uganda, Bangladesh and India (Ngonzi et al., 2013; Nessa et al., 2014; Basu et al., 2016).Comparison of Gynocular was done with standard colposcope, and biopsy as gold standard. Level of agreement with stationary colposcopy was 70.1% in Uganda. It showed 83.3% sensitivity, 23.6% specificity and 88.6% NPV in the Bangladesh study, while it demonstrated a sensitivity of 96.4% at HSIL+ threshold in India. Unlike Smart Scope^®^, Gynocular^TM^ stays outside the body of patient and hence lacks maneuverability.

*Strengths and limitations*

Unlike other devices which were tested on known screen-positive women, the SS test was carried out without prior knowledge of cervical health status of participants. In the present study, the SS test was used primarily as a screening test and not for triaging of screened positive women. In this study, the images captured after application of acetic acid, as well as, Lugol’s iodine were taken into consideration for grading of the lesion. Data obtained through this study has a good representation of confirmed CIN and cancerous cases (20 out of 509). The SS test was able to identify all the confirmed CIN I+ cases taking the sensitivity, as well as, NPV to 100%. The study participants belonged to urban and rural areas, as well as, from different socio-economical strata. We believe that the SS test has the potential to reduce the turnaround time and waiting period which is integral to screening with the Pap test. The high sensitivity and NPV of SS test as against those of Pap and NE test may help to reduce the referral rate and burden on colposcopists in tertiary settings.

SS test showed a very low specificity and PPV. To overcome these limitations, a machine learning model is being developed for auto-assessment of lesions. Further efforts are in place to improve the image quality and magnification of the camera. The study was conducted in the OPD of a tertiary care hospital and all the screening and interpretations were done by a single expert. We plan to involve the expertise of more than one observers in future trials. Also, we plan to conduct a similar study in rural settings where the device will be used by a primary health care worker. 

In conclusion it can be said that the SS test is a simple and highly sensitive screening test which gives immediate results requiring a low level of infrastructure.

## Footnotes

Smart Scope^®^ visual screening test (SS test), Naked eye visual screening test (NE test)
